# Vertical Alignment of Anisotropic Fillers Assisted by Expansion Flow in Polymer Composites

**DOI:** 10.1007/s40820-022-00909-2

**Published:** 2022-08-02

**Authors:** Hongyu Niu, Haichang Guo, Lei Kang, Liucheng Ren, Ruicong Lv, Shulin Bai

**Affiliations:** 1 School of Materials Science and Engineering, HEDPS/Center for Applied Physics and Technology, Key Laboratory of Polymer Chemistry and Physics of Ministry of Education, Peking University, Beijing, 100871 People’s Republic of China; 2 Peking University Nanchang Innovation Institute, 14#1-2 Floor, High-level Talent Industrial Park, High-Tech District, Nanchang City, 330224 Jiangxi Province People’s Republic of China

**Keywords:** Boron nitride (BN), Silicone, Expansion flow, Vertical alignment, Through-plane thermal conductivity

## Abstract

Orientation control of anisotropic one-dimensional (1D) and two-dimensional (2D) materials in solutions is of great importance in many fields ranging from structural materials design, the thermal management, to energy storage. Achieving fine control of vertical alignment of anisotropic fillers (such as graphene, boron nitride (BN), and carbon fiber) remains challenging. This work presents a universal and scalable method for constructing vertically aligned structures of anisotropic fillers in composites assisted by the expansion flow (using 2D BN platelets as a proof-of-concept). BN platelets in the silicone gel strip are oriented in a curved shape that includes vertical alignment in the central area and horizontal alignment close to strip surfaces. Due to the vertical orientation of BN in the central area of strips, a through-plane thermal conductivity as high as 5.65 W m^−1^ K^−1^ was obtained, which can be further improved to 6.54 W m^−1^ K^−1^ by combining BN and pitch-based carbon fibers. The expansion-flow-assisted alignment can be extended to the manufacture of a variety of polymer composites filled with 1D and 2D materials, which can find wide applications in batteries, electronics, and energy storage devices.

**Supplementary Information:**

The online version contains supplementary material available at 10.1007/s40820-022-00909-2.

## Highlights

- The vertically aligned structures of anisotropic fillers (using 2D boron nitride (BN) platelets as a proof-of-concept) in composites are constructed assisted by the expansion flow.
- BN platelets in composites are aligned in a curved shape that includes vertical alignment in the central area and horizontal alignment close to strip surfaces.
- The shape of expansion channels affects the vertical orientation order and the through-plane thermal conductivity of composites.

**Supplementary Information:**

The online version contains supplementary material available at 10.1007/s40820-022-00909-2.

## Graphical abstract

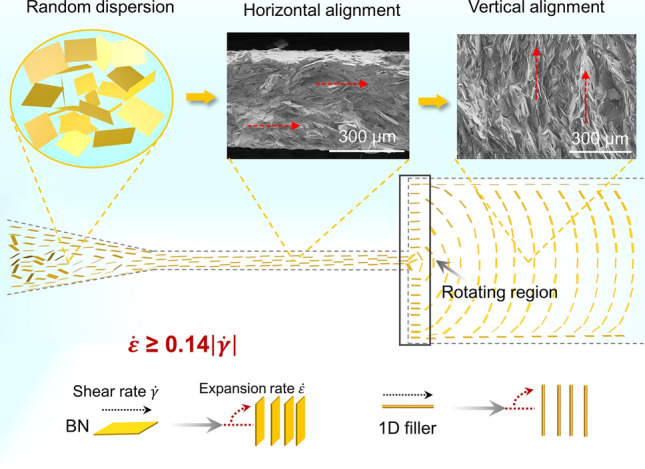

## Introduction

One-dimensional (1D) materials (e.g., carbon fiber and carbon nanotube) and two-dimensional (2D) materials (e.g., graphene, boron nitride (BN), and molybdenum disulfide) with outstanding electrical, thermal, optical, and mechanical properties have become increasingly important in applications of energy, sensors, and electronics fields [1, 2]. For extending their practical applications, these anisotropic fillers are usually assembled into polymer matrix due to the low cost, low density, flexibility, and diverse processibility of polymers [3, 4]. Owing to the outstanding axial or in-plane properties of these fillers, it is often required to align them in a certain direction along which the composites exhibit superior performance [5, 6]. Therefore, longitudinal assembly of anisotropic fillers in polymer composites is a critical pathway for producing advanced composites with extraordinary electrical, thermal, and mechanical functionalities [7, 8].

Shear stress-assisted alignment is a common strategy for alignment regulation of anisotropic particles in material processing, such as the doctor blade [9], three-dimensional (3D) printing [10, 11], or wet spinning methods [12]. It is demonstrated that shear stress in the flow field enables the alignment of sheets or fibers along the flow direction [13, 14]. In the previous studies, horizontal alignment of 2D sheets with high orientation degree has been achieved assisted by shear stress, yet well vertical alignment is still challengeable [15–17]. Expansion flow has attracted much attention since anisotropic fillers would align perpendicular to the flow direction when passing from a narrow channel to a wide channel [18–22]. The earliest study by M. Vincent in 1985 showed the fibers orientation inversion in the flow through a divergent channel [23]. It was demonstrated that fibers were mostly oriented perpendicular to the flow lines near the channel center, whereas they were oriented parallel to them near the channel wall. Recently, a vertically aligned porous graphene oxide (GO) foam structure was constructed by pumping the GO liquid crystal dispersion from a contracted channel to a sudden expansion channel [24, 25]. The high expansion stress generated by the sudden expansion can rotate GO sheets from parallel to perpendicular to the flow direction, and the vertical alignment is maintained downstream throughout the remaining part of the channel [26]. This expansion-flow-assisted alignment is inspiring for the scalable and continuous production of polymer composites with vertically aligned structures.

Hexagonal boron nitride (h-BN), also named white graphene, shows excellent mechanical strength, high in-plane thermal conductivity (monolayer BN ~ 751 W m^−1^ K^−1^), good electric insulation (bandgap 5.20–5.97 eV) properties, which exhibits potential applications for thermally conductive and insulating materials [27]. The simple incorporation of h-BN into polymer usually leads to a relatively low through-plane thermal conductivity (TC) due to the large interface thermal resistance (ITR) and anisotropic TC of h-BN [28–30]. Constructing continuous thermal pathways is an efficient strategy to decrease ITR and enhance the through-plane TC [31–33]. Therefore, various methods, such as cutting-stacking, magnetic/electric field-assisted alignment, and 3D printing, were studied to form vertical BN assembly to enhance the through-plane TC [34–40]. For example, Guo et al. reported a stacking-cutting method to obtain vertical BN assembly relying on the transformation of alignment direction, and the as-prepared BN silicone composites showed a through-plane TC of 5.97 W m^−1^ K^−1^ at 60 wt% BN loading [41]. Hu et al. fabricated BN nanosheets (BNNS) rod with vertically aligned multiscale structure by 3D printing, and the TC was up to 5.65 W m^−1^ K^−1^ with 50 wt% BNNS [42]. However, these methods are usually manual work, low production rate, and low filler loading. Therefore, a more universal and scalable method is desired for the continuous production of BN composites with vertically aligned structures.

Here, the expansion-flow-assisted alignment was utilized and extended to directly assemble anisotropic fillers into vertically aligned structures in polymer composites. BN/SG inks were pumped from a narrow channel to a wide channel in a 3D-printed expansion mold. The shape of molds shows a great influence on the orientation order of BN platelets, and consequently determined the thermal conduction performance of BN/SG strips. This vertical alignment assisted by expansion flow enables continuous production of 1D and 2D fillers filled composites with vertically aligned structures, showing potential for fast, scalable, and low-cost fabrication of highly thermally conductive composites.

## Materials and Experiments

### Materials

Vinyl silicone oil (viscosity 1000 cps, two components A and B) was bought from Shanghai Guiyou New Materials Co., Ltd., China. Component A is composed of hydrogen-containing and vinyl silicone oil. Component B includes platinum complexing catalyst and vinyl silicone oil. Hexagonal boron nitride (h-BN) with different sizes were used, one is h-BN called PT110 (D50 ~ 42 μm) obtained from Momentive Tech. Corp., USA (Fig. S1a-b), and the others (30, 15, and 5 μm in lateral size) with silane coupling agent treatment were provided by Bestry Performance Materials Corp., China. For convenience, h-BN is represented by BN. Pitch-based carbon fiber (CF) (TC, ~ 600 W m^−1^ K^−1^) was provided by Liaoning NOVCARB Carbon Materials Co. Ltd.

### Preparation of Vertically Aligned BN/SG Strips

**Fig. 1:**
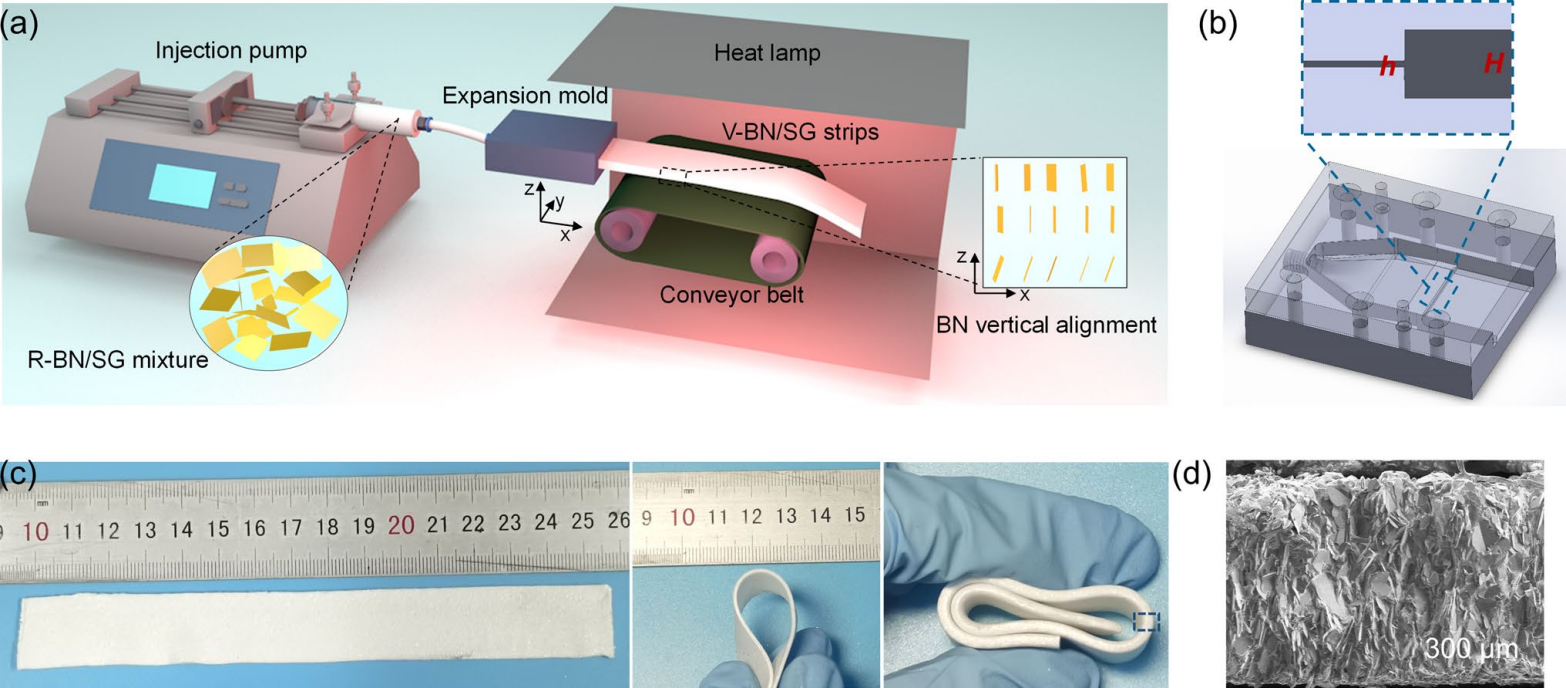
Fabrication of flexible V-BN/SG strips featuring vertically aligned BN in the central area. **a** Schematic of the continuous fabrication of V-BN/SG strips, composed of the injection pump, expansion mold, conveyor belt, and heat lamp. **b** The expansion mold designed by the Solidworks software and the inset showing changed height of microchannels from small h to large H. **c** The prepared flexible V-BN/SG strips with controllable length and thickness. **d** SEM image of 60 V-BN/SG (0.2–2) strip (0.5 mm thickness) obtained by cutting off the top and bottom skin layers

Firstly, the BN/silicone gel (SG) inks with various BN loading were prepared by a planetary centrifugal mixer (TWM-200, PCM). Typically, for BN/SG inks with a solid content of 60 wt%, 12 g of BN powders were added to 8 g silicone oil (A: B = 1:1), followed by mixing at 1000 rpm for 5 min. Then the viscous BN/SG inks were loaded in a 10 mL syringe, and the air inside the inks was eliminated. The vertically aligned BN/SG (V-BN/SG) strips were fabricated through the self-designed device (Fig. 1a), mainly including an injection pump, expansion mold, conveyer, and heat lamp. The injection pump with a maximum force of 20 kg was used to pump the viscous inks passing through the expansion microchannel. The flow speed was fixed at 2 mL min^−1^ to make sure the laminar flow pattern, matched with the conveyor speed. The extruded BN/SG strips were carried by a conveyor and cured by a heating lamp. The curing time was determined by the operating time of SG and heating temperature. The as-prepared BN composites with 60 wt% solid content are marked as the 60 V-BN/SG. For comparison, BN/SG composites with randomly dispersed BN were fabricated by directly curing the premixed BN/SG inks in a Teflon mold at 80 °C for 30 min, labeled as R-BN/SG. For evaluating the universality of the extrusion method, vertically aligned carbon fibers (V-CF/SG) were prepared by the similar extrusion procedures of the BN/SG inks. Besides, V-CF/BN/SG strips were also fabricated, in which BN and CF accounted for 50 and 10 wt%, respectively. The filler used in all composites strips is BN (PT110) except for rheological characterizations.

### Expansion Molds

The stereolithography (SLA) 3D printing method with a precision of 50 μm was used to fabricate expansion molds. The mold drawing and photographs were shown in Fig. S2. The mold is composed of a contracted channel, a narrow channel, and a wide channel. The mold is named by the height of the narrow channel (*h*) and wide channel (H) as h–H. The length of the contracted section, narrow section, and wide channel of the mold are 10, 5, and 20 mm, respectively. Figure S2c shows the picture of mold 0.2–2 (i.e., h = 0.2 mm, H = 2 mm). For visualizing the flow of BN inks in the mold, a transparent mold with h (0.4 mm) and H (2 mm) was used. The sample fabricated by the expansion mold is marked as ‘V-BN/SG (mold type)’. For example, BN strips with 60 wt% BN fabricated by the mold 0.2–2 are named as 60 V-BN/SG (0.2–2).

### Characterization

Field-emission scanning electron microscopy (FESEM, S-4800, HITACHI, Japan) was used to observe microstructures of samples. Transmission electron microscope (TEM) images were obtained by FEI Talos F200S instrument. The size distribution was measured by a laser particle size analyzer (Mastersizer3000, Malvern Panalytical, England). The 1D X-ray diffraction (XRD) was conducted by an X-pert 3 powder system operating at 40 kV with a Cu Kα radiation (*λ* = 0.154 nm). The thermal conductivity was calculated by the following equation: *k* = *α·C*_*p*_*·ρ*, where *α* is thermal diffusivity, *C*_*p*_ specific heat capacity, and *ρ* density of composites. The thermal diffusivity *α* was measured by LFA 467 HyperFlash system (NETZSCH, Germany). A density analyzer (PEAB, XS105DU, METTLER TOLEDO, Switzerland) was used to measure the density based on the Archimedes principle. The specific heat capacity was acquired by a differential scanning calorimetry (DSC, Q2000, TA Instruments) with a heating rate of 10 °C min^−1^ from − 25 to 75 °C in N_2_ flow (50 mL min^−1^).

The rheology test was conducted by the TA instrument rheometer (Advanced Rheometric Expansion System, ARES-G2, USA) with a parallel plate (diameter of 40 mm). The viscoelastic property of BN/SG inks was measured by an oscillatory time sweeping experiment by increasing frequency from 0.0001 to10 rad s^−1^ at a strain of 0.5%, 0.05%, 0.01%, and 0.01% for 50, 52, 55, and 60 wt% BN/SG inks, respectively. The apparent viscosity was tested in a shear rate range of 10^–4^ to 10^–1^ s^−1^ in the flow testing mode. The 2D wide-angle X-ray scattering (WAXS) measurement was taken on a WAXS diffractometer (Bruker D8 Discover, Germany). The wavelength of the X-ray was 0.154 nm, and the exposure time was 300 s for all samples. The Herman’s orientation factor *(f)* was calculated from fitting plots of azimuthal-integrated intensity distribution curves of X-ray patterns with Eqs. (1) and (2):

1 $$f = \frac{{3\left\langle {\cos^{2} \varphi } \right\rangle - 1}}{2}$$

2 $$\left\langle {\cos^{2} \varphi } \right\rangle = \frac{{\mathop \smallint \nolimits_{0}^{{\frac{\pi }{2}}} I\left( \varphi \right) \cos^{2} \varphi \sin \varphi d\varphi }}{{\mathop \smallint \nolimits_{0}^{{\frac{\pi }{2}}} I\left( \varphi \right) \sin \varphi d\varphi }}$$

where *φ* is the azimuthal angle and I(*φ*) is the 1D intensity distribution along with the azimuthal angle *φ* of the (002) plane [43–45].

## Results and Discussion

### Structural Analysis of the Extrusion Process Assisted by Expansion Flow

The fabrication of V-BN/SG strips includes three steps: the formulation of BN/SG inks, extrusion of BN/SG inks through the expansion channel, and in situ thermo-curing on the conveyor. Figure 1a shows the continuous production of the V-BN/SG strips enabled by the traction and fast heat curing. Figure 1b exhibits the microchannels inside the 0.2–2 mold. The narrow channel enables horizontal alignment with high order, whereas the strong expansion stress generated at the outlet of the narrow channel can rotate BN platelets toward the vertical direction. The prepared V-BN/SG strips show great flexibility (Fig. 1c). The length, thickness, and width of BN/SG strips depend on the ink volume, outlet height, and width of the mold, respectively. Figure 1d shows the well vertically aligned structure of the central area across the sample thickness. The expansion-flow-assisted alignment can be extended to other 2D materials (e.g., graphene) and 1D material (e.g., CF) for constructing vertically aligned structures, demonstrating universal applications of this method in vertical alignment control of anisotropic particles in polymer fluid (Fig. S3).

**Fig. 2:**
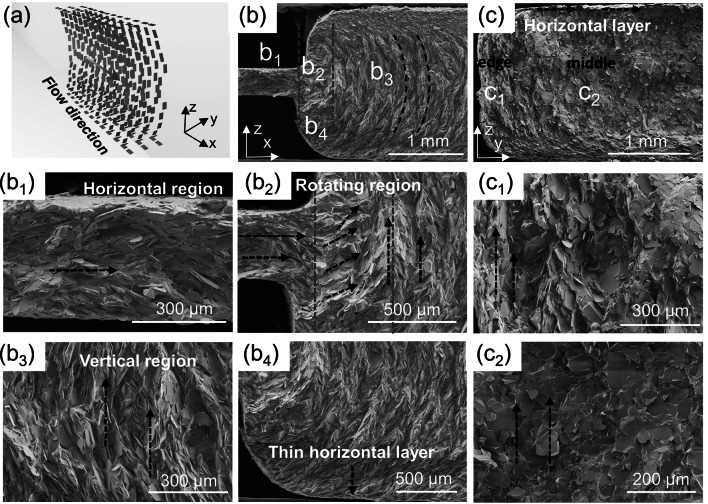
The longitudinal and transverse morphologies of 60 V-BN/SG (0.2–2) strips. **a** Schematic of BN alignment in the expansion mold. **b** Longitudinal view (*x*–*z* plane) showing the alignment rotation of BN in the expansion microchannel, divided into the horizontal region **b**_**1**_, the rotating region between two yellow lines **b**_**2**_, vertical region **b**_**3**_, the horizontal layer **b**_**4**_. **c** Transverse view (*z*–*y* plane) showing alignment difference of BN plane in the edge **c**_**1**_ and middle area **c**_**2**_

To confirm the alignment structure and rotating process of BN platelets, SEM characterization in longitudinal (*x*–*z* plane) and transverse direction (*y*–*z* plane) of samples was conducted (Fig. 2a). In general, the longitudinal SEM image (Fig. 2b) of 60 V-BN/SG (0.2–2) strips can be divided into three regions, i.e., horizontal region (*b*_1_), rotating region (*b*_2_), and vertical region (*b*_3_). In the horizontal region (*b*_1_), BN platelets were aligned parallel to the narrow channel wall due to the strong shear stress. In the rotating region (*b*_2_), the abrupt rotation of BN assisted by expansion stress is completed within ~ 500 μm away from the inlet of the wide channel. BN platelets show highly vertical alignment close to the centerline (*b*_3_), and gradual rotation to the horizontal direction close to the channel wall (*b*_4_), confirming an orientation in a curved shape (Fig. 2b). The transverse SEM image (Fig. 2c) shows that the basal plane of BN is perpendicular to the flow direction and vertically aligned along the thickness direction (*c*_2_) in the central area of the channel. However, the BN basal plane close to the side wall of the channel is parallel to the flow direction but still vertically aligned due to the shear stress (*c*_1_). The transverse SEM images also confirm the horizontally aligned layer close to the top or bottom walls (Fig. 2c, b4). The thin layer may consist of one- or two-layer aligned BN, and its thickness may depend on the additives, rheology of inks, and processing parameters.

**Fig. 3:**
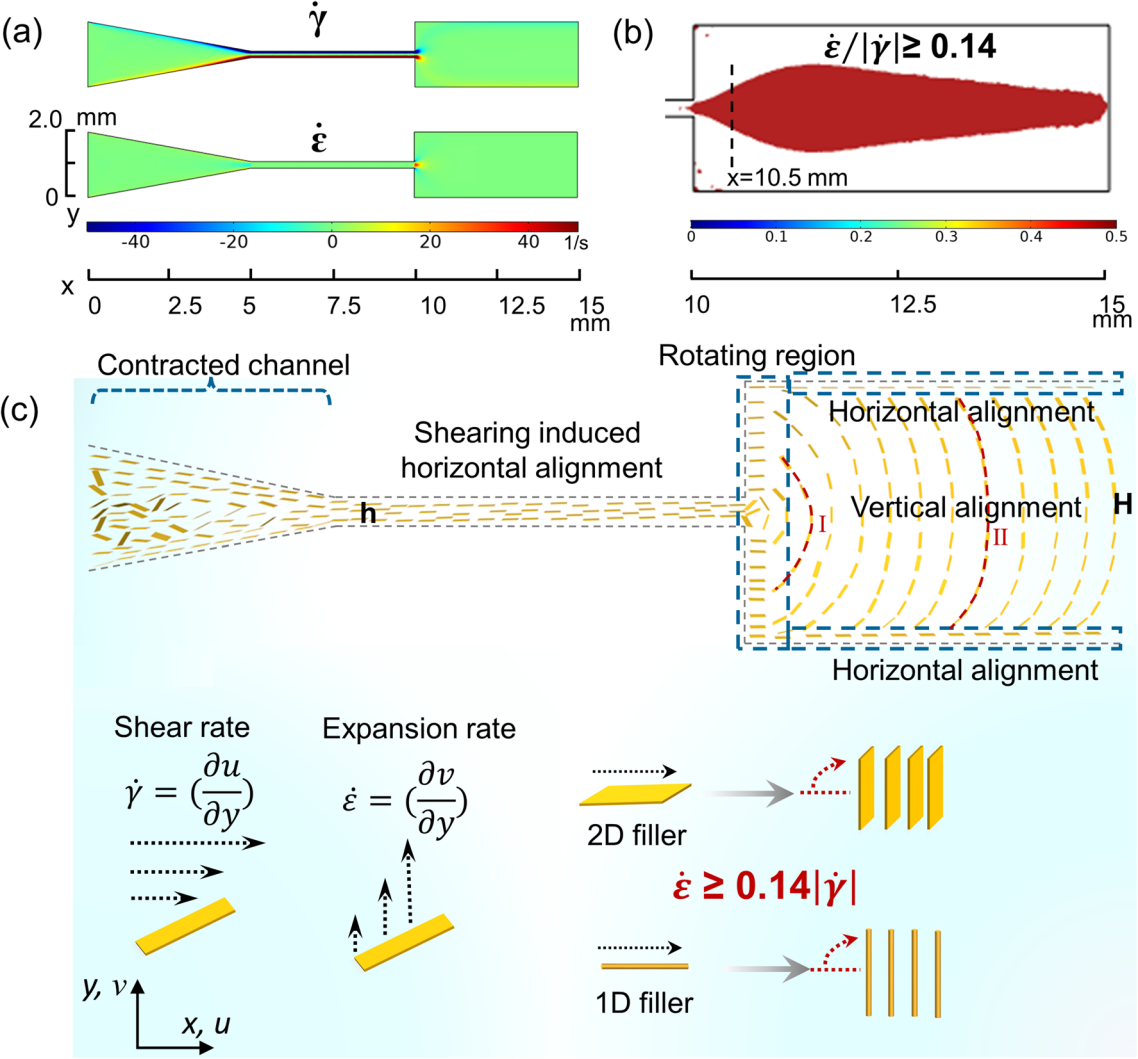
Mechanism of vertical alignment in expansion flow. **a, b** CFD computed shear rate, expansion rate, and the ratio of expansion and shear rate. **c** Illustration of the rotating process of BN platelets from parallel to perpendicular to the flow direction

For understanding the alignment process of BN in expansion flow, the velocity field (*x* and *y* components of the velocity, *u* and *v*) in the expansion channel was analyzed by the CFD simulation (Fig. S4). The shear rate $\dot{\gamma }$ and expansion rate $\dot{\varepsilon }$ defined as $\left(\frac{\partial u}{\partial y}\right)$ and $\left(\frac{\partial v}{\partial y}\right)$ are calculated by the velocity distribution. According to previous simulations and experiments on expansion-flow-assisted alignment, the expansion rate would rotate fillers toward a perpendicular direction whereas the shear rate tends to rotate fillers to a parallel direction. A relative value of expansion rate and shear rate is responsible for the filler rotation, and the region where $\dot{\varepsilon }/\left|\dot{\gamma }\right|$≥ 0.14 is chosen to represent the region with perpendicular alignment [26]. Figure 3a shows that the shear rate close to the narrow channel wall is large, which can facilitate the horizontal alignment of BN along the flow direction. The expansion rate enabling perpendicular alignment shows the maximum value at the entrance of the wide channel while the shear stress is also strong. Therefore, the ratio of expansion and shear rate (Fig. 3b) is quite crucial for predicting the rotation of 2D materials. The red region ( − 0.25 mm ≤ *y* ≤ 0.26 mm, Fig. S7) at *x* = 10.5 mm shows great agreement with the vertically aligned region in Fig. 2b. The extrusion and rotation process of BN platelets in the expansion channel is proposed in Fig. 3c. When BN platelets are flowing in the contracted and narrow channel, they are highly oriented along with the flow streamlines due to the high shear rate [46–48]. The 2D sheet geometry and large lateral size (D50 ~ 42 μm) can facilitate the alignment of BN platelets under shear stress as they flow through the narrow channel (Fig. S1a–b) [24]. Due to the large expansion rate (Fig. 2c), BN platelets would be oriented perpendicular to the flow direction at the entrance of the wide channel, and maintain the orientation as they flow downstream [26]. In particular, partial BN platelets close to channel walls would be oriented parallel to flow direction due to shear force, which is also consistent with the experiment (Fig. 2).

Velocity profiles across the channel were calculated to investigate their influence on the vertically aligned region area and alignment evolution. The Reynolds number for 60 wt% BN inks is as small as ~ 10^–7^ when the angular frequency is 100 rad s^−1^, representing a laminar stokes flow (*R*e <  < 1) in both the narrow and wide channels (Eq. (3)):

3 $${\text{Re}} = \rho \nu L/\mu$$

Here, *R*e is the Reynolds number, *ρ, ν, L,* and *μ* represent fluid density, flow speed, characteristic linear dimension, and dynamic viscosity, respectively. This laminar flow indicates that the vertically aligned structure formed at the inlet of the wide channel can be maintained downstream [10, 49]. Figure S12a shows the velocity profile at *x* = 0.1 mm and 0.5 mm is parabolic probably due to the large velocity along the centra line. However, the velocity profile at 2 mm away from the outlet of the narrow channel is non-parabolic as expected for shear-thinning fluid, representing a constant velocity in the central area and a sharp velocity decrease close to the channel walls [26]. The constant velocity in the central area is beneficial to alignment maintaining of BN platelets in the remaining part of the channel. The region with constant velocity can be tuned by the flow velocity or viscosity of inks (Fig. S6b). The calculated velocity profiles show great agreement with the curved-shape alignment (Fig. 2) and proposed extrusion process (Fig. 3c I-II). Since the extrusion process involves the transformation of horizontal to vertical alignment, the orientation order can be highly affected by the horizontal orientation degree. To facilitate horizontal alignment in the narrow channel, flat and circular channels with minimized size should be chosen for 2D filler and 1D fillers, respectively (Fig. 3c).

### Rheological Characterization of BN/SG Inks

**Fig. 4:**
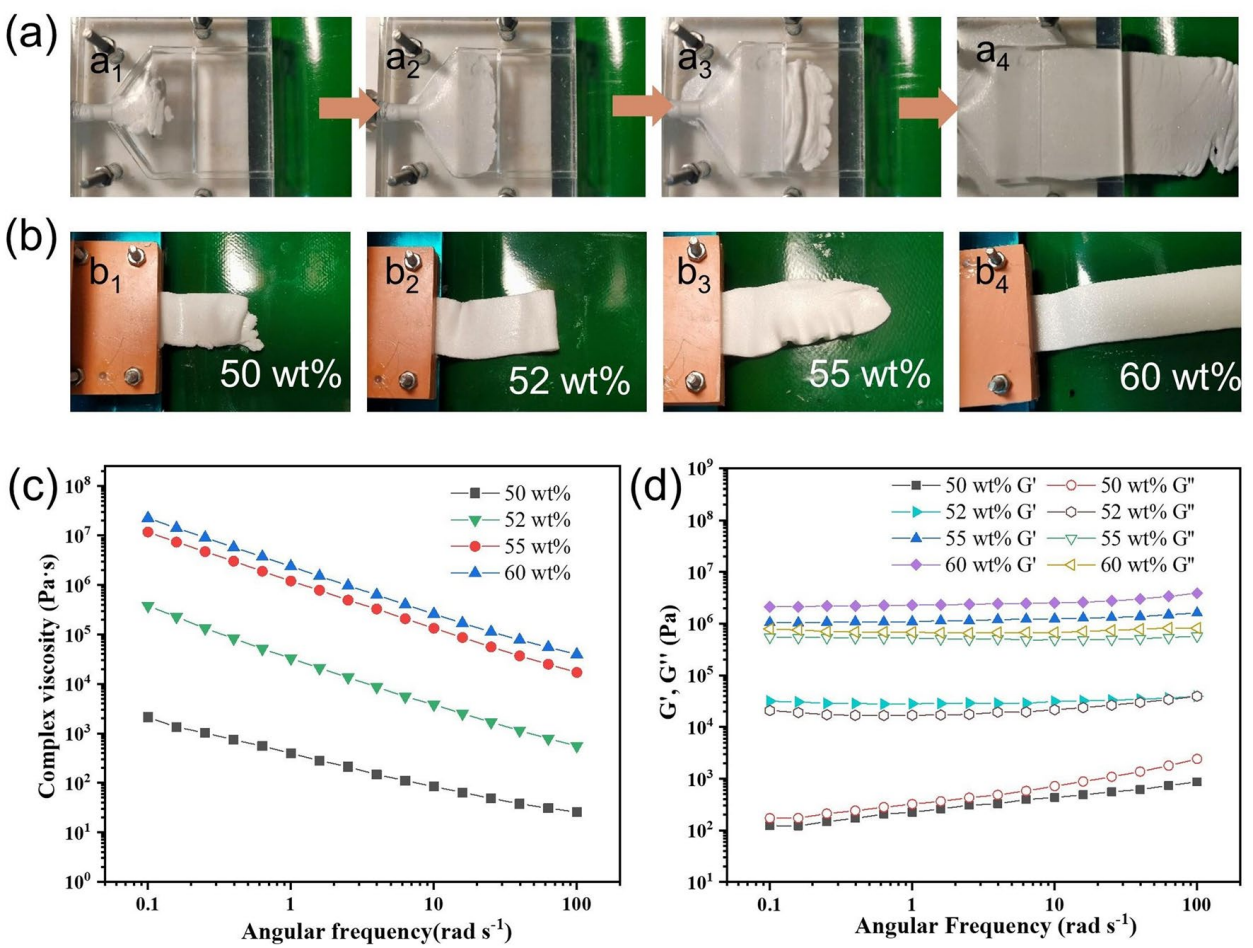
Rheological properties and processibility of BN/SG inks. **a** Digital image showing the flowing of 60 wt% BN/SG inks in the expansion mold. **b** Extruded V-BN/SG strips at different BN contents. Plots of **c** the complex viscosity and **d** storage (*G*′) and loss (*G*′′) moduli as a function of angular frequency

Rheological properties of BN/SG inks are vital for smooth extrusion of V-BN/SG strips, which relate to the properties of silicone oil, BN size, and contents [50]. A transparent mold was utilized to visualize the BN extrusion process (Fig. 4a). The BN/SG inks were observed to flow from the circular inlet of the mold and gradually to the width direction until full filling of the mold. After flowing through the narrow channel, wave-like strips were initially observed, and then continuous V-BN/SG strips were formed. To ensure the smooth extrusion and shape retention of BN/SG strips, high and low viscosity was prohibited because of the following reasons: high viscosity would result in clogging in the narrow channel of the mold; inks with low viscosity would fail to maintain the intact shape after extrusion, even leading to the collapse of structures. Digital images (Fig. 4b) show that inks with 50 wt% BN content cannot retain their shape while the edge of the strip filled with 52 wt% BN is smooth and intact. Therefore, the 52 wt% BN is considered to be the critical content for the continuous production of V-BN/SG strips in this experiment. When the BN content is further increased up to 65 wt%, the clogging would happen due to ultra-high viscosity. In addition, BN size shows a significant effect on the content range allowing for smooth extrusion of V-BN/SG strips (Fig. S8). It is interesting to note that inks filled with small-sized BN prone to form wavy strips at high content probably due to poor fluidity. The cross-sectional image of the wave-like strips indicates that the extruded BN lamina is self-stacked perpendicular to the flow direction, resulting in vertically aligned BN structures (Fig. S9).

To evaluate the ability of extrusion, rheology measurements were taken for ink with BN contents of 50, 52, 55, and 60 wt%. Figure 4c shows that inks at all BN loading demonstrate obvious shear thinning behavior, as evidenced by a significant decrease of complex viscosity with increased angular frequency from 0.1 to 100 rad s^−1^, which facilitates the flowing of highly viscous BN inks through the thin microchannel. The viscoelastic properties of BN/SG inks are essential for the shape and structure retention of extruded V-BN/SG strips. The storage (*G*′) and loss (*G*′′) moduli of BN/SG inks are given as a function of angular frequency in Fig. 4d. For 50 wt% BN/SG ink, *G*′ and *G*′′ increase as the increasing angular frequency, and a higher *G*′′ than *G*′ represents the dominance of viscous force, thus hard to retain their shape after extrusion. However, the 60 wt% BN/SG ink exhibits *G*′ plateaus at ~ 2 × 10^6^ Pa s higher than *G*′′, indicating a gel-like behavior demonstrated by previous reports [50, 51]. The gel-like inks with BN contents of 52–60 wt% would flow after a stress yield point during the extrusion process because of shear thinning. When the inks flow out of the mold, the physical entanglement of BN platelets forces them to keep the high elasticity which enables the shape and structure retention.

### Characterization of the Orientation Order

**Fig. 5:**
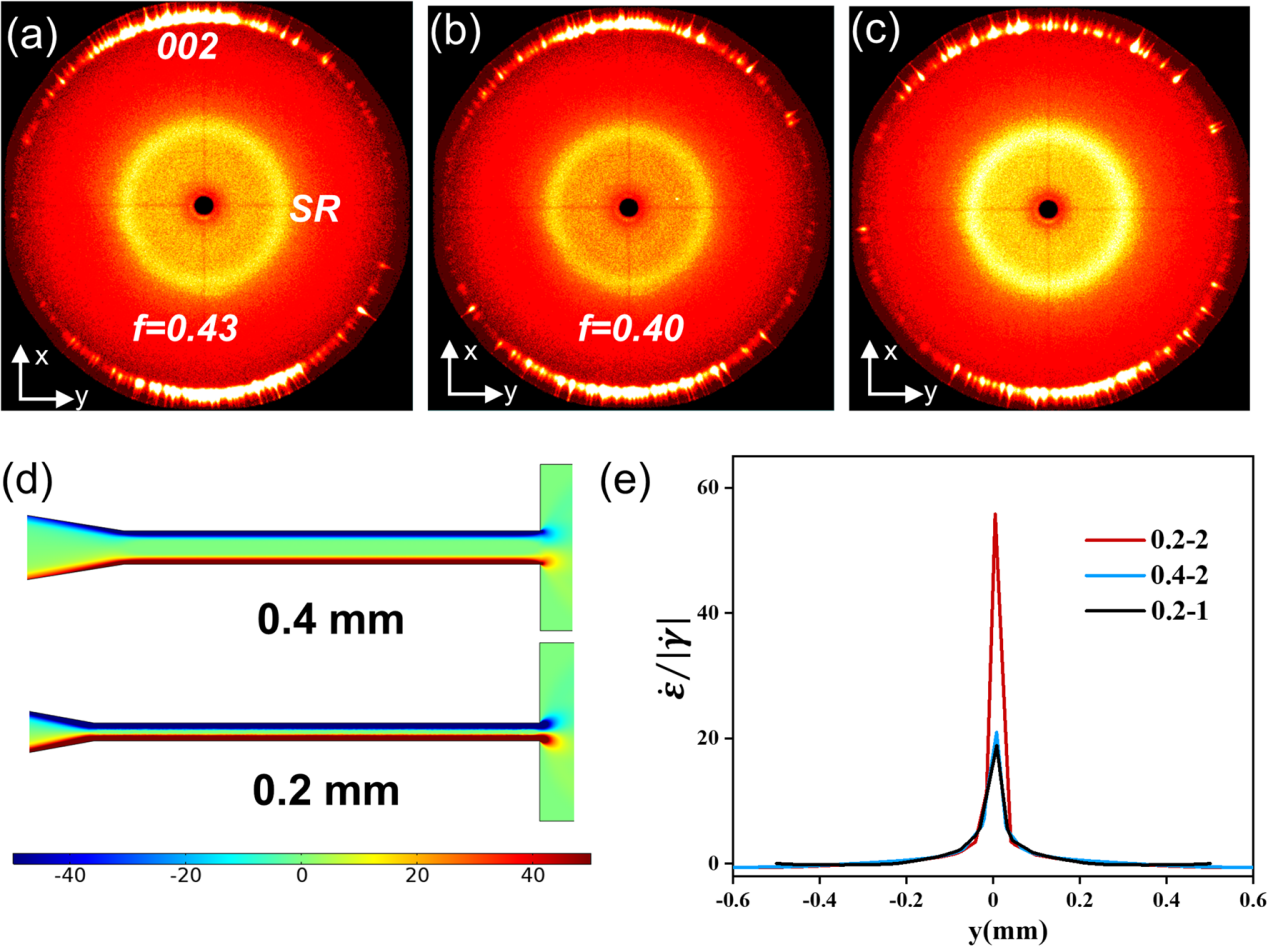
Orientation analysis and comparison of expansion molds with different shapes. 2D WAXS patterns from the *x*–*y* plane of 60 V-BN/SG strip fabricated by expansion molds, **a** 0.2–2, **b** 0.4–2, **c** 0.2–1. **d** Calculated shear rate distribution along the longitudinal narrow channel with 0.2 and 0.4 mm height. **e** Comparison of the rate ratio at *x* = 10.5 mm of these molds as a function of the location *y* in the wide channel

To figure out the influence of channel shapes on the vertically aligned structures, the orientation order of 60 V-BN/SG strips was quantitatively evaluated by 2D WAXS analysis (Fig. S10). There are three kinds of expansion mold (0.2–2, 0.2–1, 0.4–2) (Fig. S2). The thickness of measured samples is half of the initial thickness of the strip by cutting off the top and bottom surface. The powder XRD pattern (Fig. S11a) demonstrates the hexagonal crystal with a strong diffraction peak at 2*θ* = 26.8 (corresponding to the (002) plane). For the 60 V-BN/SG (0.2–2) strip, the 2D WAXS pattern of the *x*–*y* and *x*–*z* plane shows strong (002) reflection at the meridian (Figs. 5a and S10b), which disappears for the *y*–*z* plane (Fig. S10c). These results indicate that the basal plane of BN indeed aligns perpendicular to the flow direction in the expansion channel, corresponding to the morphology analysis in Fig. 2 [45]. The calculated orientation factor for the 0.2–2 mold is ~ 0.43, indicating the well vertical alignment of BN. However, the orientation factor enabled by the 0.4–2 mold is ~ 0.40, slightly lower than that of the mold 0.2–2 (Fig. 5b), which can be attributed to its lower shear rate from the narrow channel wall that aligned the BN platelets parallel to the flow direction (Fig. 5d). As discussed in Fig. 3, better horizontal orientation order of BN in the narrow channel would lead to superior vertical alignment after BN rotation. The WAXS profile of 0.2–1 mold (Fig. 5c) shows scattered diffraction spots compared to these of 0.2–2 and 0.4–2 ones, indicating its inferior orientation order. Besides, orientation parameters of 0.2–1 cannot be accurately calculated because of the low fitting coefficient of determination (Fig. S12).

It is believed that the expansion rate helps to rotate BN platelets toward a perpendicular direction while the shear rate rotates them toward a parallel direction. As the rotation of BN is finished within 0.5 mm away from the inlet confirmed by the SEM image (Fig. 2b), the rate ratio is calculated at *x* = 10.5 mm (Fig. 5e). The plot shows that the maximum value of 0.2–2 mold is larger than those of 0.4–2 and 0.2–1 ones, which may explain the optimal orientation of BN in 0.2–2 mold. For the 0.2–1 mold, the larger shear rate in the wide channel may break the well-aligned structure, resulting in inferior vertical orientation order compared to that of the 0.2–2 mold. In summary, in the rotating region, the vertical orientation order in the wide channel is positively associated with the expansion/shear rate ratio as well as the horizontal orientation order in the narrow channel. However, in the remaining part of the wide channel, the shear rate may play a major role in the particle orientation evolution [20].

### Thermo-conductive Characterization of the BN/SG Strips

**Fig. 6:**
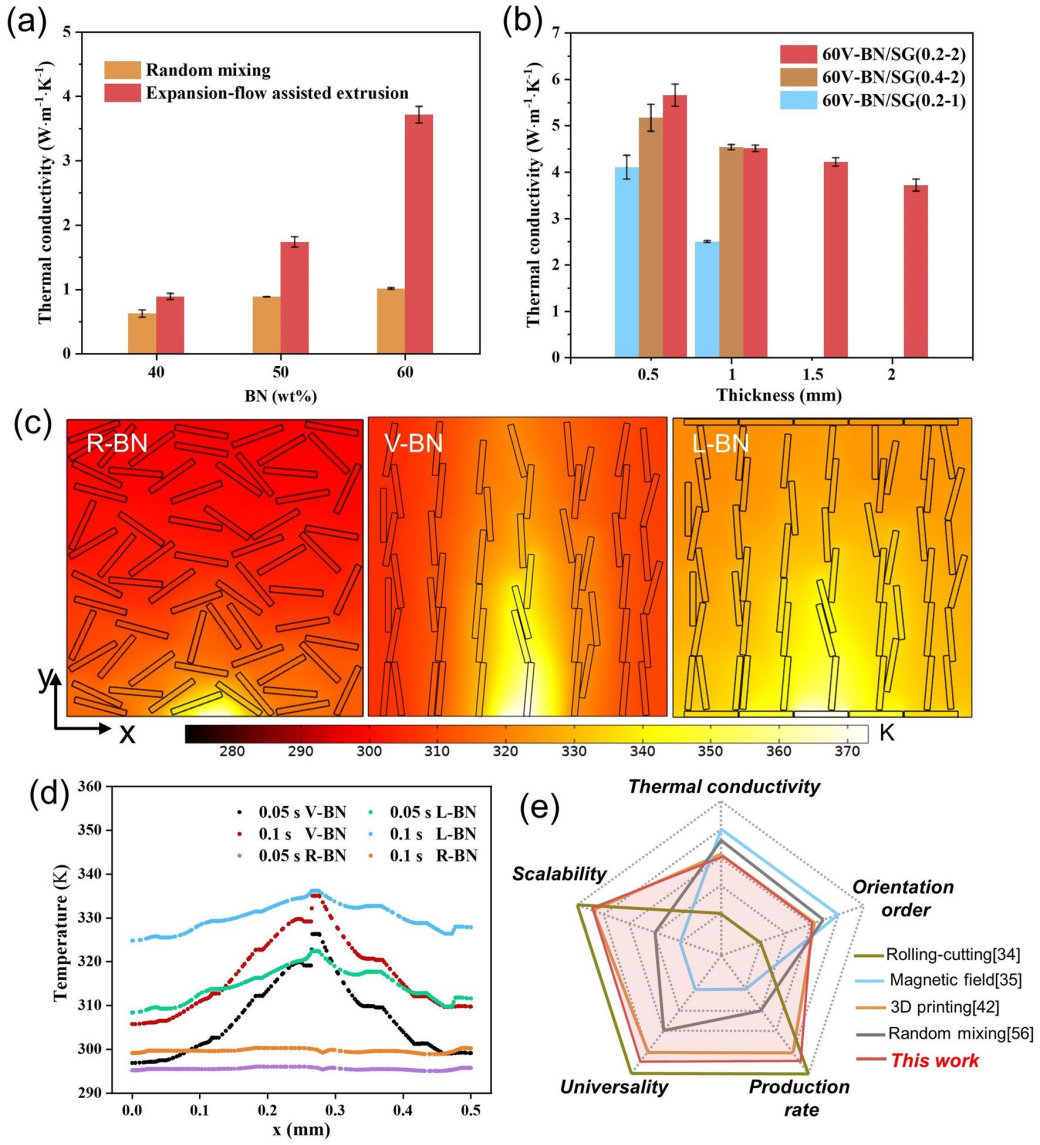
Thermal conductivity of V-BN/SG strips. **a** Through-plane thermal conductivity of BN/SG strips as a function of filler loading. **b** Through-plane thermal conductivity of V-BN/SG strips fabricated by three kinds of molds as a function of sample thickness. **c** Simulated comparison of heat transfer of randomly dispersed BN (R-BN), vertically aligned BN (V-BN), and ladder-structure BN (L-BN) assembly heated by a localized heat source for 0.1 s. **d** Temperature distribution at the half-height of these three simulated boxes. **e** Qualitative comparison of our work with reported ones regarding the BN composites fabrication [34, 35, 42, 56]

The BN/SG strips composed of vertically aligned BN in the center and thin horizontally aligned layers on the top and bottom surfaces, featuring the continuous and scalable production, are promising for thermal management of electronic devices [28, 52–54]. Figure 6a shows the through-plane thermal conductivity (TC) of both V-BN and R-BN composites gradually increases with increasing BN contents while values for expansion-flow-assisted extrusion with the 0.2–2 mold are always larger than that for random mixing. The 2 mm-thickness 60 V-BN/SG (0.2–2) shows through-plane TC up to 3.80 W m^−1^ K^−1^, which greatly exceeds that of randomly mixing BN composite having only 1.01 W m^−1^ K^−1^. The higher through-plane TC value of V-BN/SG (0.2–2) can be attributed to efficient heat conduction along with the vertically aligned BN in the thickness direction (Fig. 6c). However, the thermal conduction of 60 V-BN/SG (0.2–2) at 2 mm thickness is suppressed because of the large thermal resistance close to the top and bottom sides. Therefore, the through-plane TC is further increased to 5.65 W m^−1^ K^−1^ by cutting off the bottom and top surface layers until the thickness is reduced to 0.5 mm (Fig. 6b). The finite element simulation (software *Comsol5.5*) was carried out to compare the heat transfer process inside samples with different BN assemblies heated by a localized heat source at a transient state (Fig. S16). It demonstrates that the vertically aligned BN shows better thermo-conductive performance than randomly dispersed BN according to the higher temperature of V-BN at 0.05 and 0.1 s (Fig. 6d). The simulated heat conduction image and temperature curve of ladder-structure BN show a more uniform temperature distribution than V-BN due to the heat spread of horizontally aligned BN, indicating that V-BN/SR samples before cutting off the top and bottom surface layers can avoid local overheating and improve the effective contact thermal conduction [55].

In terms of the critical role of the BN orientation order on the thermo-conductive properties of composites, the influence of mold shapes on the through-plane TC is also investigated (Fig. 6b). The results show that there is a minor difference in through-plane TC of 60 V-BN/SG strips with 0.2–2 and 0.4–2 molds. However, the through-plane TC of 60 V-BN/SG (0.2–2) is slightly higher than that of 60 V-BN/SG (0.4–2) at 0.5 mm thickness because of the higher orientation order enabled by the 0.2–2 mold along the centra line as discussed in Fig. 5. For 60 V-BN/SG (0.2–1), the through-plane TC is only 2.51 W m^−1^ K^−1^ (1 mm thick) much lower than that of other molds although this value is improved to 3.89 W m^−1^ K^−1^ at the thickness of 0.5 mm. The through-plane TC data are provided in Table S2. When 10 wt% CF is added, the through-plane TC of V-BN/SG (0.2–2) (1mm) increases from 4.55 to 6.54 W m^−1^ K^−1^ due to higher TC of CF, and synergistic effect of CF and BN platelets in thermal conduction. Therefore, it is believed that the expansion-flow-assisted method can achieve higher TC by employing fillers with higher TC (such as graphene and carbon fiber). Furthermore, compared with other methods for fabricating BN-filled polymer composites from five aspects: TC, orientation order, scalability, universality, and production rate, our method has great potential for scalable fabrication of BN or other 1D/2D materials filled polymer composites with advanced performances (Fig. 6e).

**Fig. 7:**
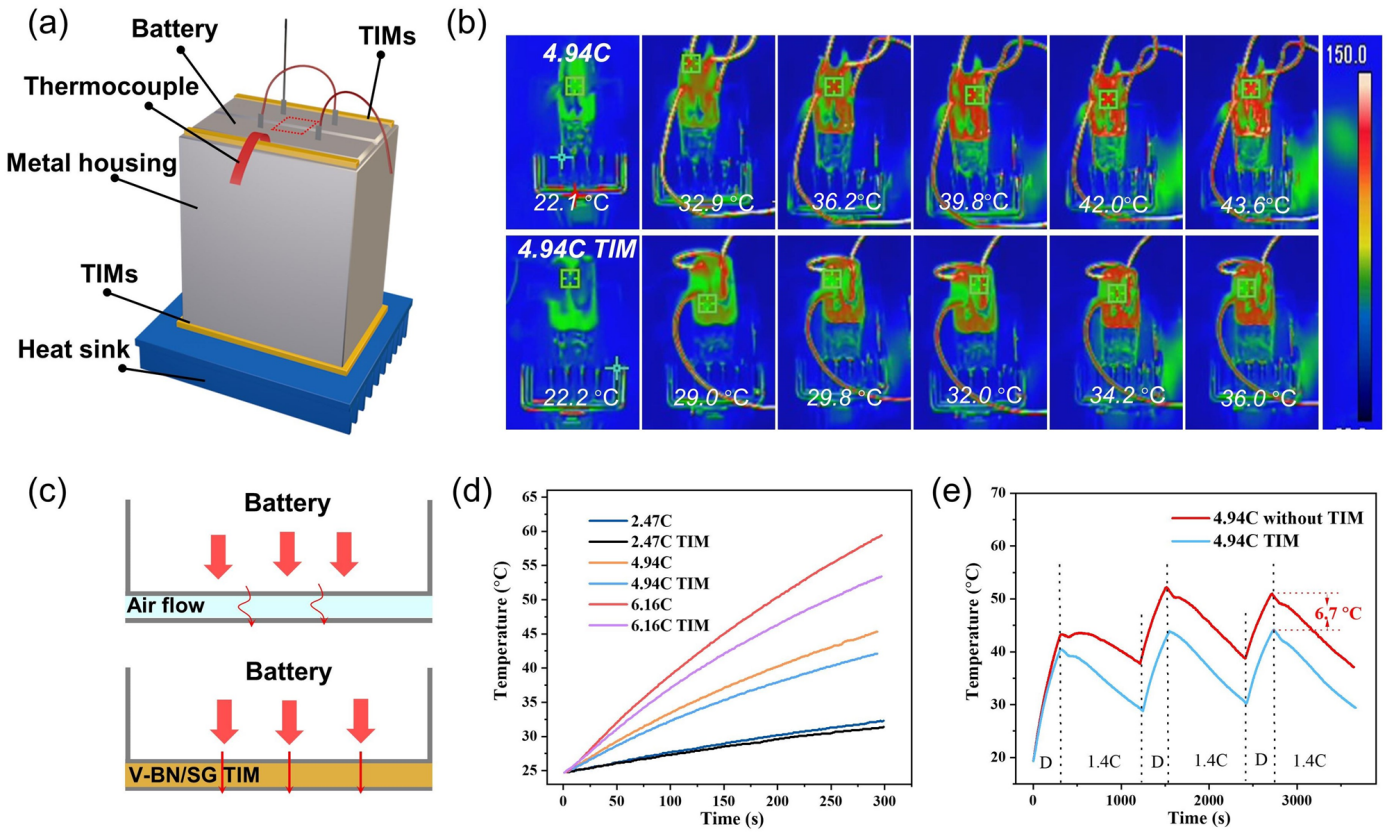
Battery thermal dissipation performance of 60 V-BN/SG (0.2–2) strips. **a** Schematic illustration of the TIMs temperature measurement system in the battery setup. **b** IR images of the top surface of batteries with and without V-BN/SG strips as TIMs under 4.94C discharging rate at room temperature. **c** Schematic of TIMs used to bridge the battery and metal housing. Temperature revolution of the battery surface measured by a thermocouple as a function of time **d** under 2.74C, 4.94C, 6.16C discharging rate, and **e** during three charging (1.4C)-discharging (4.94C) cycles

The thermo-conductive performance of 60 V-BN/SG (0.2–2) was evaluated by a battery setup. As shown in Fig. 7a, two lithium-ion batteries (3.7 V, 3000 mAh) were connected in series and wrapped with iron foil. The 60 V-BN/SG (0.2–2) strip as TIMs with a thickness of 1 mm was put into the gap between the battery and iron foil, followed by pressing to make compact contact between TIMs and battery, as well as between TIMs and iron foil. The thermocouple was put between the battery and TIMs to measure the temperature of the battery side surface. To control the battery temperature below the warning temperature (60 °C), a bottom graphitic heat sink and TIMs were used to facilitate the heat dissipation of the battery pack. The discharging process was conducted by using electrical resistance with the value of 1 Ω, 0.5 Ω, or connecting two resistors in parallel, and the calculated discharging rates were ~ 2.74C, 4.94C, and 6.16C, respectively (Fig. S15). The IR images show that temperatures of the top surface of the battery pack equipped with TIMs are always lower than those without TIMs during the discharging (4.94C) process, indicating faster heat transfer from the battery to the metal housing (Fig. 7b). The surface temperature was extracted from the red square zone in the center of battery packing. As the discharging rate increases, the temperature difference of the battery side surface increases from 0.9 °C (2.47C), 3.2 °C (4.94C) to 5.9 °C (6.16C) due to the high TC of TIMs compared with that of the air (0.02 W m^−1^ K^−1^) (Fig. 7c–d). For the discharging (4.94C)-charging (1.4C) cycles (Fig. 7e), the temperature increases during the discharging process and decreases in the charging process due to the low charging rate. The maximum temperature in the second cycle is larger than that of the first cycle because of the heat accumulation. The temperature difference of the third cycle is ~ 6.7 °C, indicating the distinctive thermal dissipation capacity of the battery pack assisted with 60 V-BN/SG (0.2–2) TIMs. Furthermore, the compression test of V-BN/SG strips shows that the compressive stress is ~ 1.53 MPa at 50% compressive strain, meaning the great flexibility and resilience of TIMs (Fig. S14). Overall, the simple, universal, expansion-flow-assisted method allows us to design TIMs with highly efficient thermal-dissipation performance and flexibility, showing potential applications in electronics cooling.

## Conclusion

In summary, this work demonstrates a universal and scalable expansion-flow-assisted method for tuning the vertical alignment of 1D and 2D materials in polymer composites. The expansion stress induces the alignment inversion from the horizontal to the vertical direction, confirmed by CFD simulation. The developed alignment in a curved shape is not only beneficial to improving the through-plane TC but also favorable for avoiding local overheating. As a result, a high through-plane TC up to 5.65 W m^−1^ K^−1^ is achieved by cutting off the horizontally aligned BN layers, which can be further improved by employing fillers with higher thermal conductivity (such as graphene, pitch-based carbon fibers) or combining 1D and 2D fillers. The V-BN/SG strips used as TIMs in the battery pack demonstrate great heat dissipation capability, indicating potential applications in electronics cooling. The expansion-flow-assisted alignment not only provides a new and universal method for alignment control of anisotropic 1D and 2D materials in polymer composites but also gives an insight into the combination of traditional processing techniques (screw extruding, injection molding, 3D printing, wet-spinning, etc.) with expansion flow.

## Supplementary Information

Below is the link to the electronic supplementary material.

Supplementary file1 (PDF 1562 KB)
