# Supplementary material for: Vertical Alignment of Anisotropic Fillers Assisted by Expansion Flow in Polymer Composites
Source: Nanomicro Lett. 2022 Aug 2;14:153. doi: 10.1007/s40820-022-00909-2 (PMC9346047; doi:10.1007/s40820-022-00909-2)
Supplement: Supplementary file 1 — Supplementary file1 (PDF 1562 KB) [file 40820_2022_909_MOESM1_ESM.pdf]

Supporting Information for

Vertical Alignment of Anisotropic Fillers Assisted by Expansion Flow in Polymer Composites

Hongyu Niu[1], Haichang Guo[1], Lei Kang[1], Liucheng Ren[1], Ruicong Lv[1], Shulin Bai[1, 2, *]

[1] School of Materials Science and Engineering, HEDPS/Center for Applied Physics and Technology, Key Laboratory of Polymer Chemistry and Physics of Ministry of Education, Peking University, Beijing 100871, P. R. China

[2] Peking University Nanchang Innovation Institute, 14#1-2 Floor, High-level Talent Industrial Park, High-tech District, Nanchang City, Jiangxi Province 330224, P. R. China

*Corresponding author. E-mail address: slbai@pku.edu.cn (Shulin Bai)

Supplementary Figures

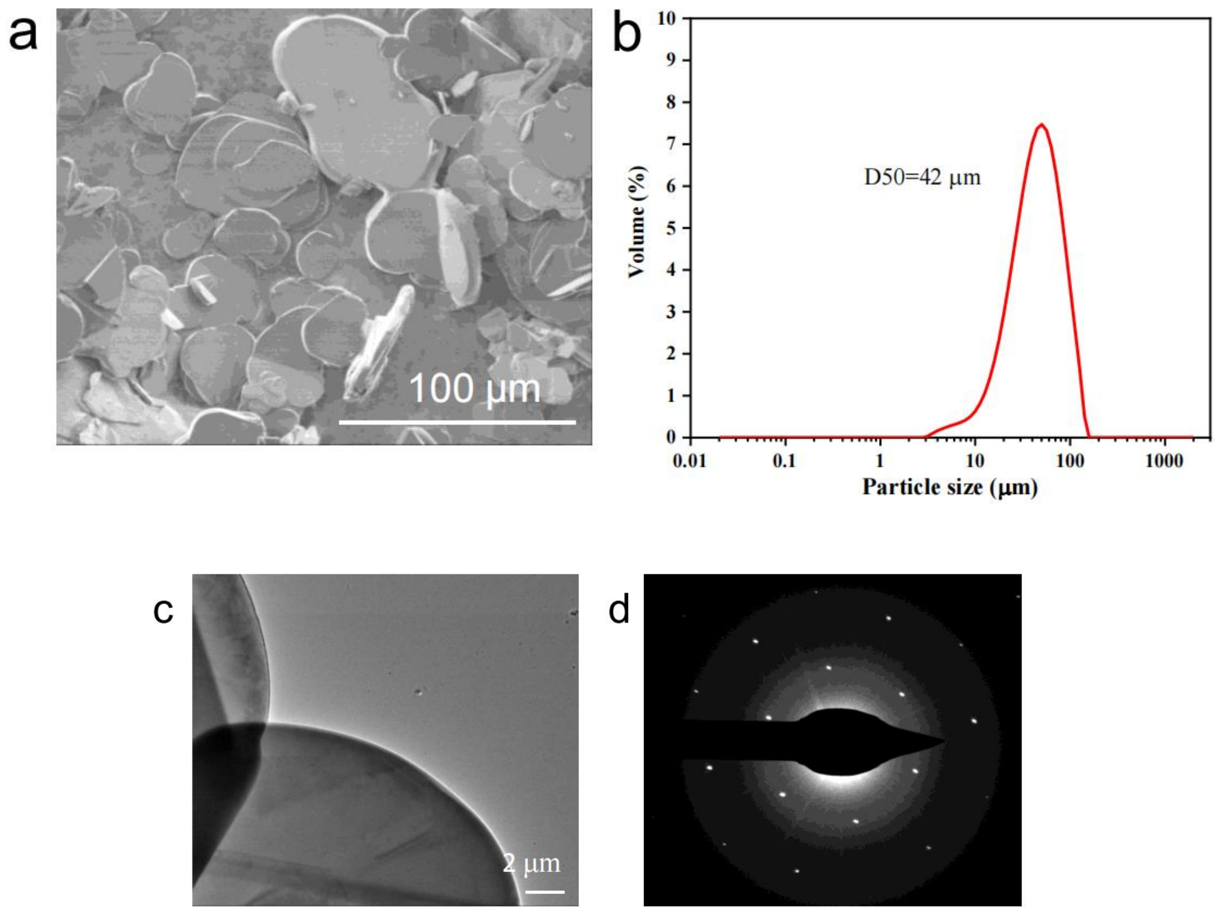

Fig. S1 Size and structure characterization of h-BN particles (PT110), **(a)** SEM image, **(b)** size distribution, **(c)** TEM image, **(d)** selected area electron diffraction (SAED)

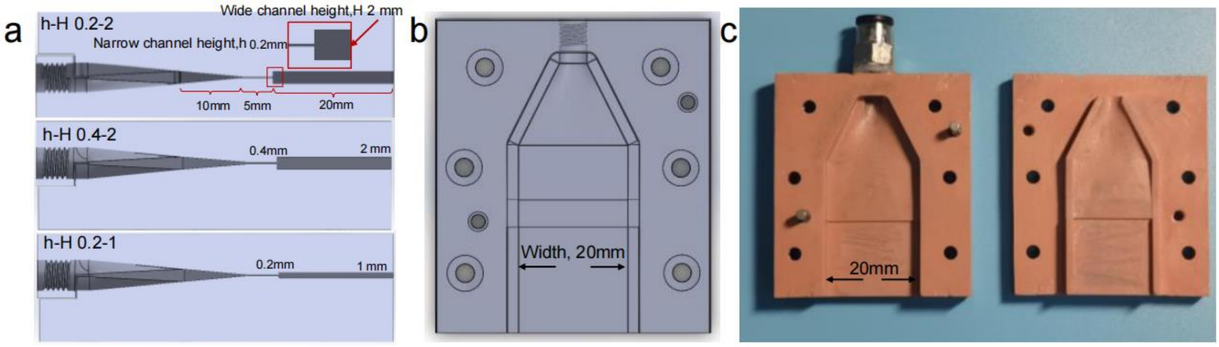

Fig. S2 The lateral view **(a)** and top view **(b)** of the mold drawing. **(c)** Photograph of the mold (0.2-2) including positive and negative molds fabricated by the 3D printing technique

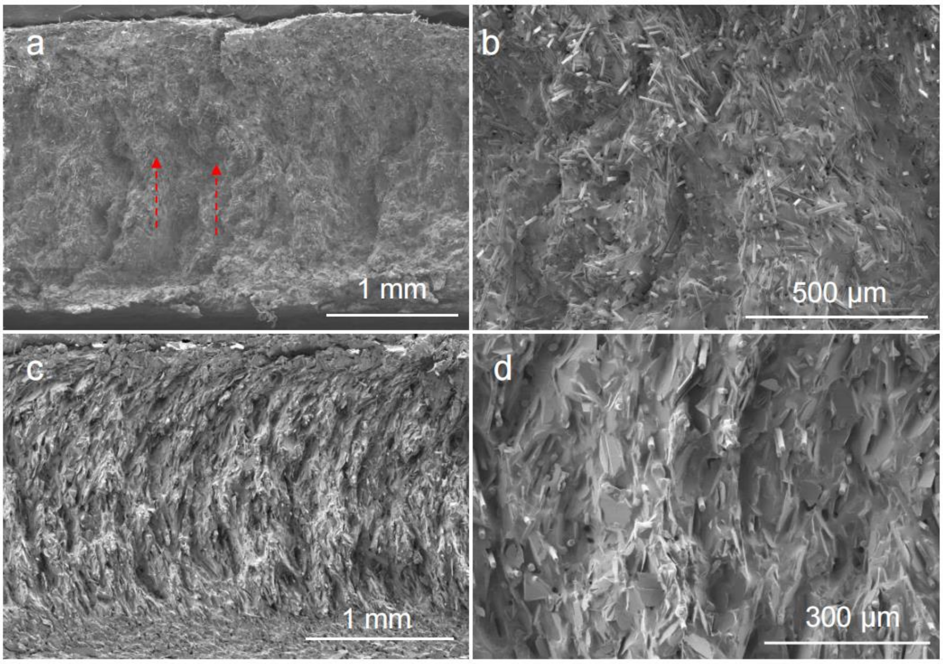

Fig. S3 Cross-sectional SEM images of the prepared strips filled with, **(a, b)** 30 wt% CF by 0.4-2.4 mold, **(c, d)** 10 wt% CF and 50 wt% BN by the 0.2-2 mold

CFD simulation

The computational fluid dynamics (CFD) analysis was conducted by the COMSOL 5.5 software by calculating the steady, single-phase and laminar flow in the expanded mold [S1, S2]. Figure S4 shows the geometry of 2D longitudinal section of the 0.2-2 mold. The relative tolerance of 10^{-3} was set as the convergence criterion in solving the continuity and momentum equations. Due to the non-Newtonian behavior of BN inks, the steady viscosity was measured as a function of shear rate from 10^{-4} to 10^{-1}. As shown in Fig. S5, the Carreau model was used to obtain the relevant parameters, including zero-shear viscosity (μ_0), time constant (λ), infinite-shear viscosity (μ_∞), and power-law index (n). These parameters listed in Table S1 were input as the fluid property considering the shear-thinning behavior. The inlet velocity with variable speed from 0.0006 to 0.0256 m s^{-1}, zero pressure outlet, and a non-slip condition on other walls were used.

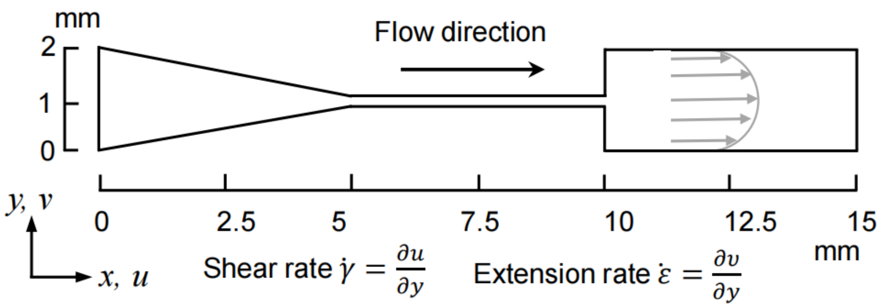

Fig. S4 The geometry of the longitudinal section of the expanded mold (0.2-2) in CFD simulation

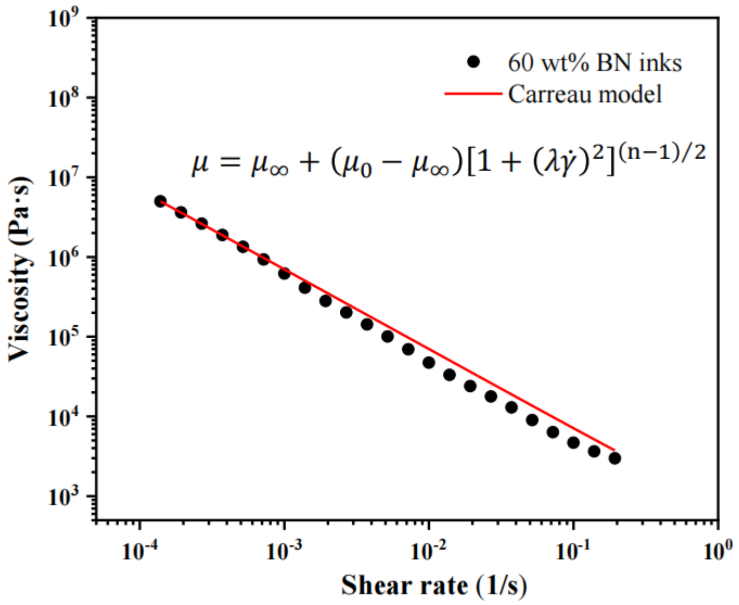

Fig. S5 The plot of viscosity as a function of shear rate and the fitting curve by the Carreau model

Table S1 Material properties used in the simulation

CFD Parameters	Non-Newtonian
Flow rate ml/min	1.5
Flow speed, m/s	6.25 10^{-4}
Zero-shear viscosity, Pa ·s	3.41 10^{7}
Infinite-shear viscosity, Pa ·s	121.7
Internal relaxation time, s	48774
Power-law exponent	0.0242
Density, Kg·m $^{-3}$	1480

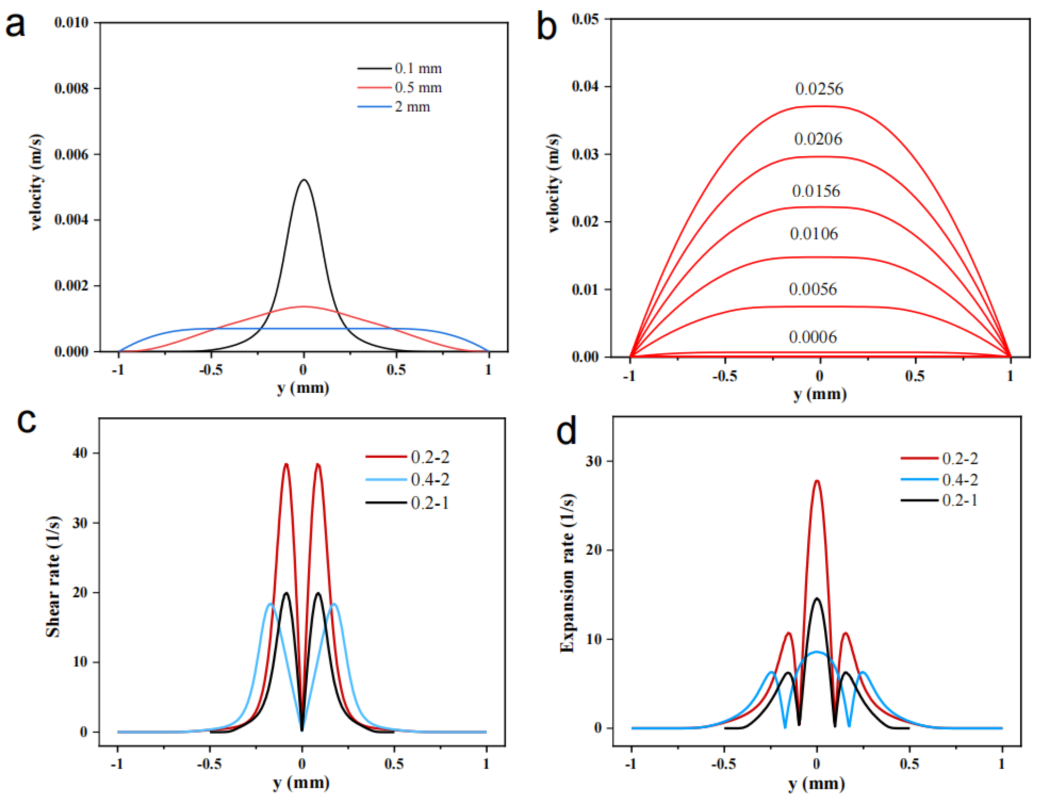

Fig. S6 Velocity field of 60 wt% BN/SG inks calculated by CFD simulation. **(a)** Comparison of velocity distribution at x= 10.1, 10.5, 12 mm. **(b)** Comparison of calculated velocity profile at x=12 mm with flow velocity from 0.0006 to 0.0256 m s^{-1}. Comparison of the calculated shear rate **(c)** and expansion rate **(d)** at x=10.1 mm, for different mold shapes

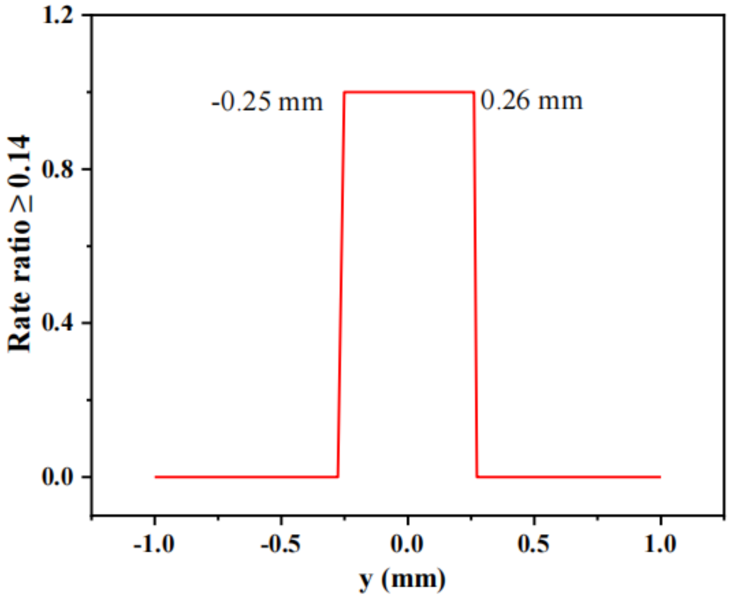

Fig. S7 The calculated rate ratio higher than 0.14 with the location along y-axis

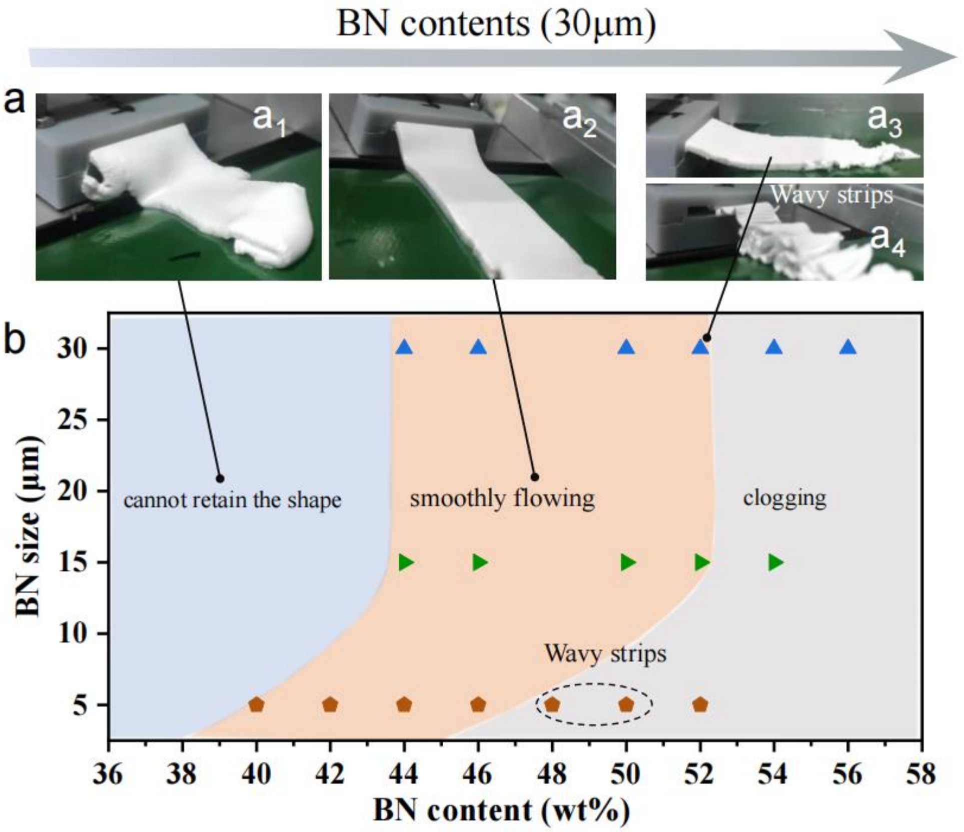

Fig. S8 (a) Uncured BN/SG strips filled with different contents, (a_1) 40 wt% (30 μm), (a_2) 50 wt% (30 μm), (a_3) 55 wt% (30 μm), (a_4) 48 wt% (15 μm). **(b)** Diagram for extrusion ability of inks with various BN sizes and contents

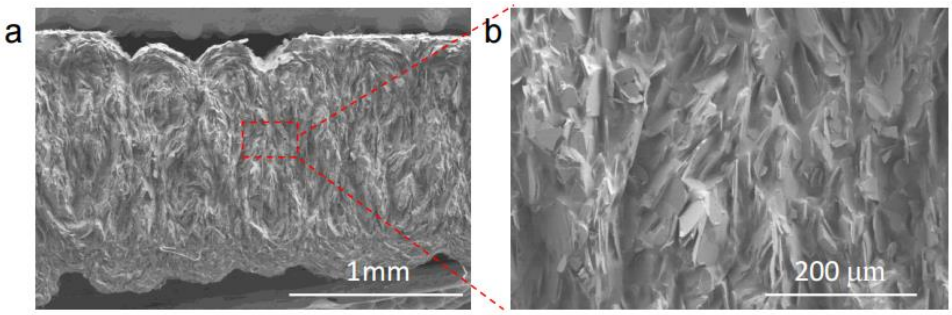

Fig. S9 Cross-sectional SEM images of the extruded wavy-like strips

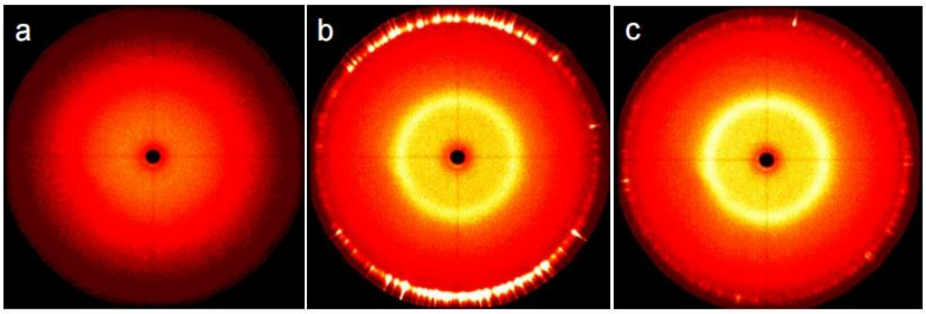

Fig. S10 2D WAXS patterns of, **(a)** pure SG, **(b)** side plane and **(c)** front plane of 60V-BN/SG (0.2-2) strip

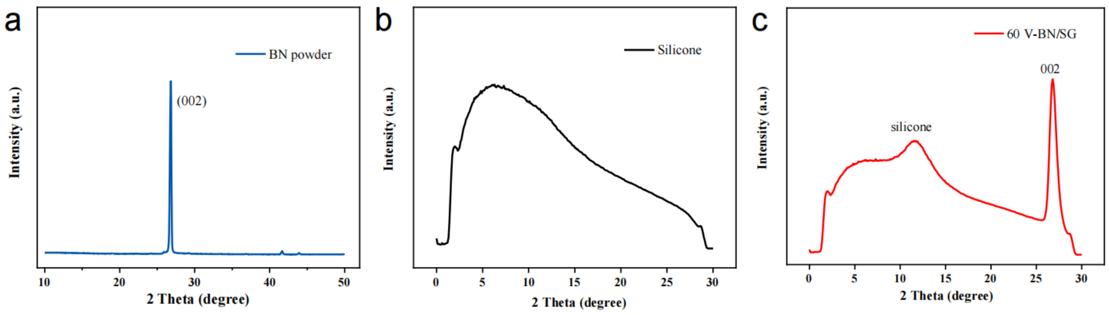

Fig. S11 (a) The powder X-Ray diffraction (XRD) of h-BN powder (PT110). Integration of WAXS patterns of **(b)** pure SR, **(c)** 60V-BN/SG (0.2-2) strip (the x-y plane)

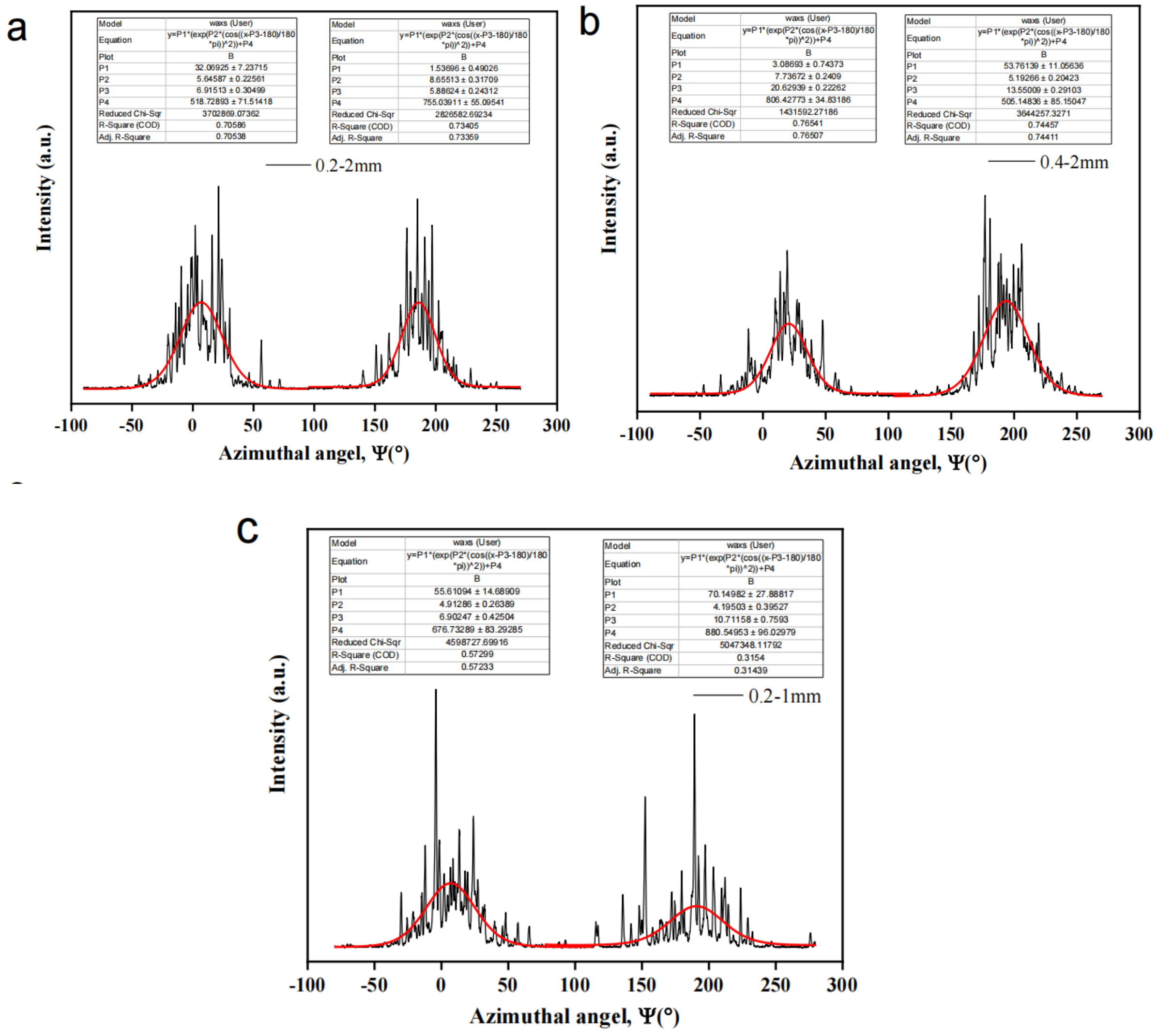

Fig. S12 The azimuthal angel (φ) plots and fitting curves of the x-y plane of strips by, **(a)** 0.2-2mm, **(b)** 0.4-2, **(c)** 0.2-2 molds

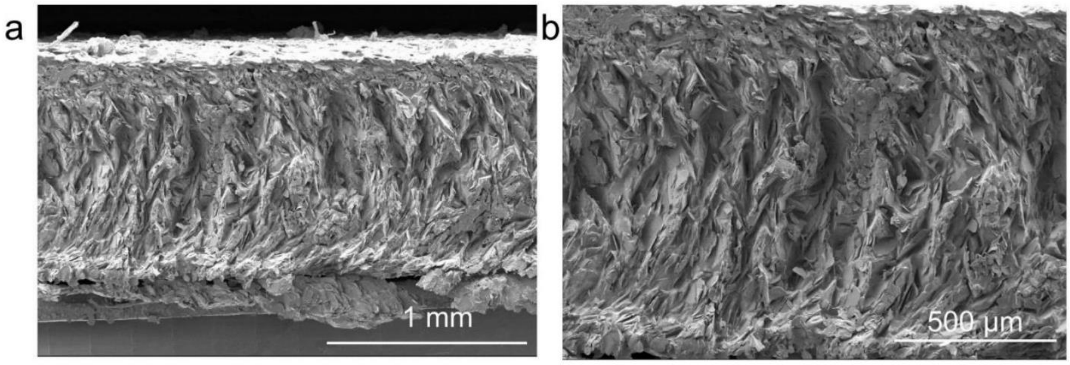

Fig. S13 The cross-sectional SEM images of the 60V-BN/SG (0.2-1) strip

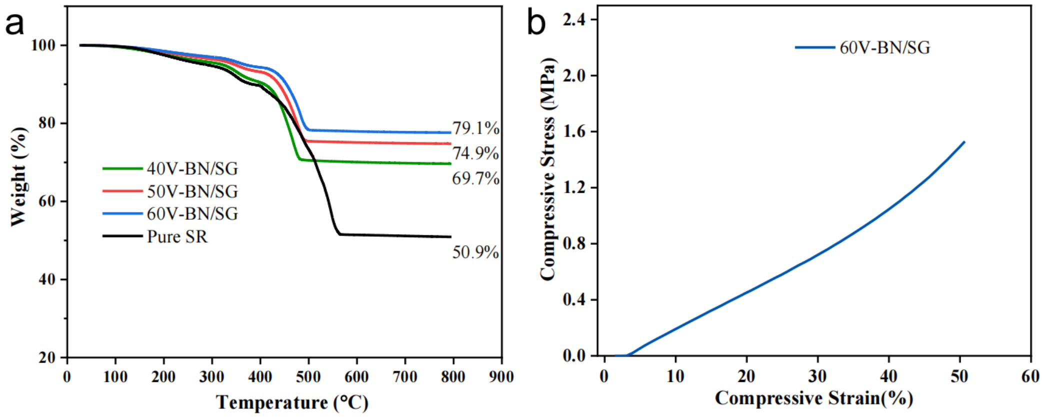

Fig. S14 (a) The TG curve of V-BN/SG (0.2-2) strips. **(b)** The compressive property of the 60V- BN/SG (0.2-2) strip

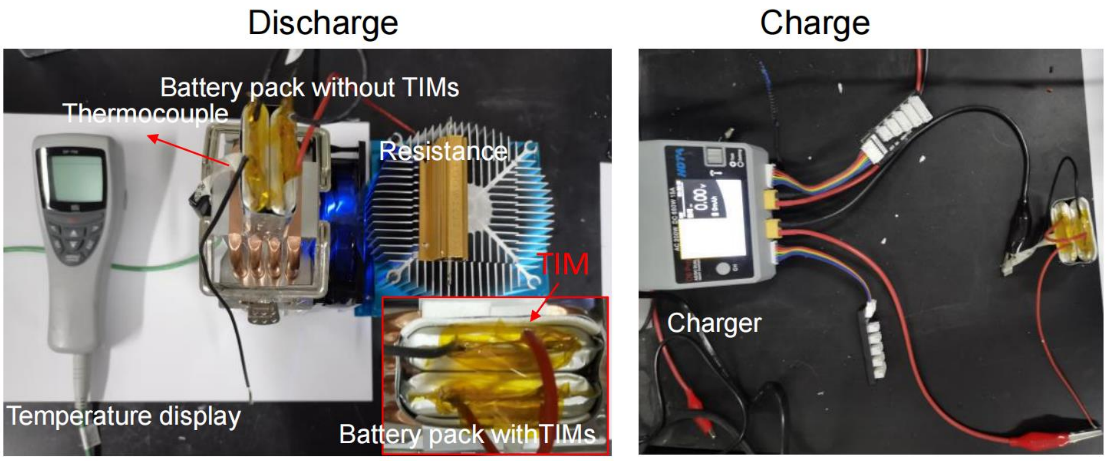

Fig.S15 Photographs of discharging and charging setup of the battery pack with BN strips as TIMs

Finite element simulation of heat conduction:

The finite element simulation was carried out by the COMSOL 5.5 software to compare the heat conduction capability of three BN assembled structures. Fig. S16 shows the simulative models and coordinate systems of BN sheets in composites with randomly dispersed, vertically aligned and ladder-like aligned structures. The calculated coordinate systems enabled the definition of anisotropic thermal conductivity of BN. Localized heat source (0.05 mm length) with a constant temperature of 100 °C was applied to the bottom of the simulation box (0.5×0.5 mm^2). The boundary condition of top surface was external natural convection with an ambient temperature (25 °C), and others were thermally insulated. The thermal conductivity of silicone rubber and BN were set to be 0.2 and 600 (in-plane) /30 (through-plane) W m^{-1} K^{-1}, respectively (Fig. S16)) [S3, S4].

a

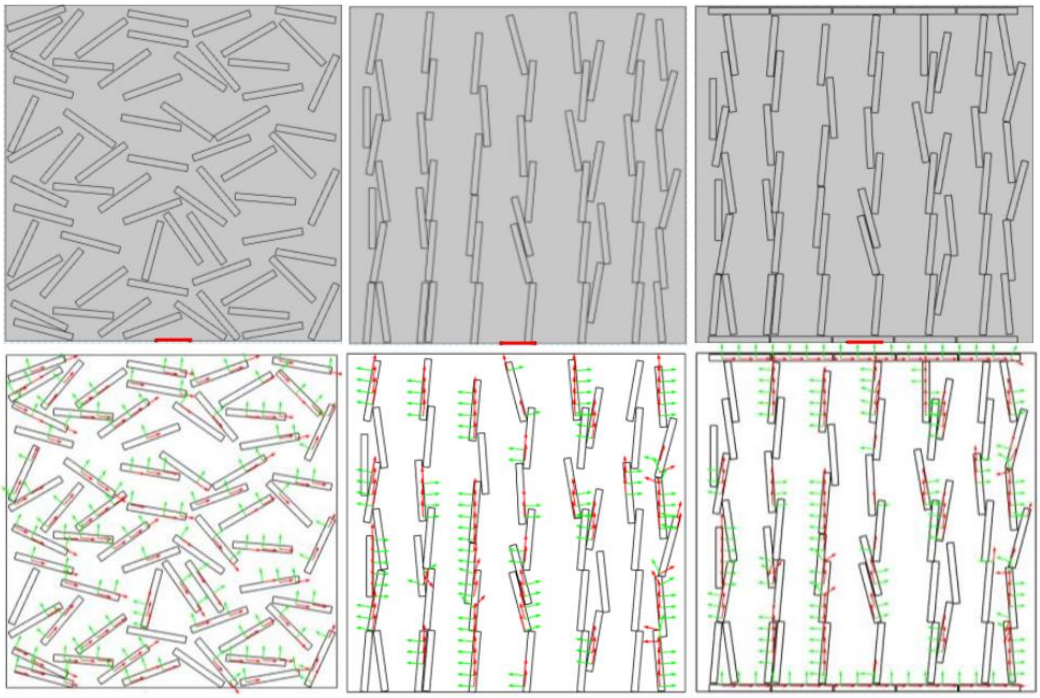

b

Parameters	SG	BN	
Thermal conductivity (W· m^{-1}·K^{-1})	0.2	In-plane	Through-plane
		600	30
Specific heat capability(J/(g ·°C)	1300	700	
Density(kg/m^3)	1	2.29	

Fig. S16 (a) Finite element simulation models (the top three images) and calculated coordinate system of each BN sheet in composites with randomly dispersed, vertically aligned and ladder-like aligned BN structures from left to right. **(b)** major parameters of SG and BN in the simulation analysis

Table S2 The calculated thermal conductivity of samples

Samples	Thickness (mm)	α ($mm^2\ s^{-1}$)	ρ ($g\ cm^{-3}$)	Cp ($J\ g^{-1}\ K^{-1}$)	TC ($W\ m^{-1}\ K^{-1}$)
40R-BN/SG	2	0.49	1.271	1.195	0.74
50R-BN/SG	2	0.676	1.362	1.113	1.02
60R-BN/SG	2	0.777	1.494	1.128	1.31
40V-BN/SG(0.2-2)	2	0.726	1.269	1.277	1.18
50V-BN/SG (0.2-2)	2	1.646	1.383	1.129	2.57
60 V-BN/SG (0.2-2)	2	2.253	1.484	1.138	3.80
60V-BN/SG (0.2-2)	1.5	2.536	1.482	1.138	4.28
60V-BN/SG (0.2-2)	1	2.704	1.480	1.138	4.55
60V-BN/SG (0.2-2)	0.5	3.453	1.482	1.138	5.82
60V-BN/SG (0.4-2)	1	2.661	1.491	1.124	4.50
60V-BN/SG (0.4-2)	0.5	2.675	1.473	1.124	4.43
60V-BN/SG (0.2-1)	1	1.495	1.49	1.128	2.51
60V-BN/SG (0.2-1)	0.5	2.485	1.516	1.128	4.25
50V-BN/CF/SG (0.2-2)	2	3.369	1.488	1.107	5.55
50V-BN/CF/SG (0.2-2)	1.5	3.399	1.484	1.107	5.58
50V-BN/CF/SG (0.2-2)	1	3.999	1.477	1.107	6.54

Table S3 Through-plane thermal conductivity of BN, BNNS based composites measured by LFA

Materials	Method	Loading	Through-plane TC ($W\ m^{-1}\ K^{-1}$)	References
BNNS/HDPE	Injection molding	23.2 vol%	1.34	[S5]
PS@BN	Hot pressing	33.3 wt%	0.94	[S6]
PP/6wt%PF@B N	Hot-pressing	40 wt%	3.85	[S7]
BNNS/BNMS/ epoxy	Direct mixing	30 wt%	1.148	[S8]
Spherical BN/PDMS	Direct mixing	50 wt%	2.32	[S9]
BNNS/epoxy	Bidirectional freezing	15 vol%	6.07	[S10]
3D BN/epoxy	Ice-templating	34 vol%	4.42	[S11]
3D BN/epoxy	NH_4HCO_3 templating	60 vol%	6.11	[S12]
BN/epoxy	Magnetic field	60 vol%	7.28	[S13]
BNNS/PS	Foaming assembly	30 wt%	1.28	[S14]
V-BN/SR	Stacking-cutting	60 wt%	5.4	[S15]
BN/MVQ	Rolling-cutting	60 wt%	6.3	[S16]
BN rod	3D printing	50 wt%	5.65	[S17]
BN/TPU	3D printing	60 wt%	~6	[S18]
V-BN/SG	This work	60 wt%	5.65	

Supplementary References

[S1] M. Trebbin, D. Steinhauser, J. Perlich, A. Buffet, S.V. Roth et al., Anisotropic particles align perpendicular to the flow direction in narrow microchannels. PNAS **110**(17), 6706-6711 (2013). https://doi.org/10.1073/pnas.1219340110

[S2] D.I. Jeong, A. Jain, D.W. Oh, Increasing perpendicular alignment in extruded filament by an orifice embedded 3D printing nozzle. Virtual. Phys. Prototyp. **17**(1), 1-18 (2021). https://doi.org/10.1080/17452759.2021.1980935

[S3] Q. Yan, F.E. Alam, J. Gao, W. Dai, X. Tan et al., Soft and self-adhesive thermal interface materials based on vertically aligned, covalently bonded graphene nanowalls for efficient microelectronic cooling. Adv. Funct. Mater. **31**(36), 2104062 (2021). https://doi.org/10.1002/adfm.202104062

[S4] K. Wu, D. Liu, C. Lei, S. Xue, Q. Fu, Is filler orientation always good for thermal management performance: a visualized study from experimental results to simulative analysis. Chem. Eng. J. **394**, 124929 (2020). https://doi.org/10.1016/j.cej.2020.124929

[S5] M. Hamidinejad, A. Zandieh, J.H. Lee, J. Papillon, B. Zhao et al., Insight into the directional thermal transport of hexagonal boron nitride composites. ACS Appl. Mater. Interfaces **11**(44), 41726-41735 (2019). https://doi.org/10.1021/acsami.9b16070

[S6] R. Wang, H. Cheng, Y. Gong, F. Wang, X. Ding et al., Highly thermally conductive polymer composite originated from assembly of boron nitride at an oil-water interface. ACS Appl. Mater. Interfaces **11**(45), 42818-42826 (2019). https://doi.org/10.1021/acsami.9b15259

[S7] X. Li, C. Li, X. Zhang, Y. Jiang, L. Xia et al., Simultaneously enhanced thermal conductivity and mechanical properties of PP/BN composites via constructing reinforced segregated structure with a trace amount of BN wrapped PP fiber. Chem. Eng. J. **390**, 124563 (2020). https://doi.org/10.1016/j.cej.2020.124563

[S8] L. Zhao, L. Yan, C. Wei, Q. Li, X. Huang et al., Synergistic enhanced thermal conductivity of epoxy composites with boron nitride nanosheets and microspheres. J. Phys. Chem. C **124**(23), 12723-12733 (2020). https://doi.org/10.1021/acs.jpcc.0c01377

[S9] L. Ren, X. Zeng, R. Sun, J.B. Xu, C.P. Wong, Spray-assisted assembled spherical boron nitride as fillers for polymers with enhanced thermally conductivity. Chem. Eng. J. **370**,166-175 (2019). https://doi.org/10.1016/j.cej.2019.03.217

[S10] J. Han, G. Du, W. Gao, H. Bai, An anisotropically high thermal conductive boron nitride/epoxy composite based on nacre-mimetic 3D network. Adv. Funct. Mater. **29**(13), 1900412 (2019). https://doi.org/10.1002/adfm.201900412

[S11] J. Hu, Y. Huang, Y. Yao, G. Pan, J. Sun et al., Polymer composite with improved thermal conductivity by constructing a hierarchically ordered three-dimensional interconnected network of BN. ACS Appl. Mater. Interfaces **9**(15), 13544-13553 (2017). https://doi.org/10.1021/acsami.7b02410

[S12] X. Xu, R. Hu, M. Chen, J. Dong, B. Xiao et al., 3D boron nitride foam filled epoxy composites with significantly enhanced thermal conductivity by a facial and scalable approach. Chem. Eng. J. **397**, 125447 (2020). https://doi.org/10.1016/j.cej.2020.125447

[S13] A. Gurijala, R.B. Zando, J.L. Faust, J.R. Barber, L. Zhang et al., Castable and printable dielectric composites exhibiting high thermal conductivity via percolation-enabled phonon transport. Matter **2**(4), 1015-1024 (2020). https://doi.org/10.1016/j.matt.2020.02.001

[S14] W. Zhou, Y. Zhang, J. Wang, H. Li, W. Xu et al., Lightweight porous polystyrene with high thermal conductivity by constructing 3D interconnected network of boron nitride nanosheets. ACS Appl. Mater. Interfaces **12**(41), 46767-46778 (2020). https://doi.org/10.1021/acsami.0c11543

[S15] Y. Xue, X. Li, H. Wang, F. Zhao, D. Zhang et al., Improvement in thermal conductivity of through-plane aligned boron nitride/silicone rubber composites. Mater. Des. **165**, 107580 (2019). https://doi.org/10.1016/j.matdes.2018.107580

[S16] Z. Yin, J. Guo, X. Jiang, Significantly improved thermal conductivity of silicone rubber and aligned boron nitride composites by a novel roll-cutting processing method. Compos. Sci. Technol. **209**, 108794 (2021). https://doi.org/10.1016/j.compscitech.2021.108794

[S17] Z. Liang, Y. Pei, C. Chen, B. Jiang, Y. Yao et al., General, vertical, three-dimensional printing of two-dimensional materials with multiscale alignment. ACS Nano **13**(11), 12653–12661 (2019). https://doi.org/10.1021/acsnano.9b04202

[S18] H. Guo, H. Niu, H. Zhao, L. Kang, Y. Ren et al., Highly anisotropic thermal conductivity of three-dimensional printed boron nitride-filled thermoplastic polyurethane composites: effects of size, orientation, viscosity, and voids. ACS Appl. Mater. Interfaces **14**(12), 14568-14578 (2022). https://doi.org/10.1021/acsami.1c23944
